# Living Alone Increases the Risk of Hypertension in Older Chinese Adults: A Population-Based Longitudinal Study

**DOI:** 10.1093/geroni/igad071

**Published:** 2023-07-03

**Authors:** Xiang Wang, Xiangyang Yuan, Bin Xia, Quan He, Wei Jie, Miao Dai

**Affiliations:** Department of Cardiology, Jiujiang No. 1 People’s Hospital, Jiujiang, Jiangxi, China; Department of Cardiology, Jiujiang No. 1 People’s Hospital, Jiujiang, Jiangxi, China; Department of Geriatrics, Jiujiang No. 1 People’s Hospital, Jiujiang, Jiangxi, China; Medical Records Department, Jiujiang No. 1 People’s Hospital, Jiujiang, Jiangxi, China; Department of Geriatrics, Jiujiang No. 1 People’s Hospital, Jiujiang, Jiangxi, China; Department of Geriatrics, Jiujiang No. 1 People’s Hospital, Jiujiang, Jiangxi, China

**Keywords:** Aging, Hypertension, Living alone, Older adult

## Abstract

**Background and Objectives:**

Cross-sectional studies have suggested a potential association between living alone and hypertension risk, but longitudinal evidence remains limited. We aimed to investigate the correlation between living alone, alterations in living arrangements, and hypertension risk among older adults utilizing a population-based longitudinal design.

**Research Design and Methods:**

The study included 8 782 older adults (≥65 years) without hypertension from the Chinese Longitudinal Healthy Longevity Survey. Participants were surveyed during the 2008 and 2011/2012 waves and were subsequently followed up in the next wave. Hypertension was defined as systolic blood pressure ≥140 mmHg and/or diastolic blood pressure ≥90 mmHg, or a self-reported diagnosis of hypertension by a physician. Cox proportional hazards model was used to explore the association between living alone and hypertension. Additionally, we analyzed how switching living arrangements during the follow-up period affects hypertension.

**Results:**

During a median follow-up of 2.8 (1.7–3.0) years, 2 750 hypertension events occurred. Compared with living with family, the hazard ratio (HR) (95% confidence interval [CI]) of living alone was 1.19 (1.06–1.33) for hypertension. Similarly, persisting in living alone during follow-up increased the risk of hypertension compared to continuing to live with family (HR 1.24; 95% CI: 1.06–1.45). Compared to married participants who continued to live with family, widowed/divorced participants who transitioned from living with family to living alone experienced a higher risk of hypertension (HR 1.21; 95% CI: 1.00–1.47). Stratified analyses showed that living alone was only associated with an increased hypertension risk for participants aged >80, men, and rural residents.

**Discussion and Implications:**

Living alone at baseline or persisting in living alone during follow-up correlated with increased hypertension risk. Divorced or widowed individuals who transitioned from living with family to living alone were still at risk. These results indicate that social support and living arrangements may be important in preventing hypertension in older adults.

**Translational Significance:** Living alone, either at the baseline or persisting during follow-up, was linked to an increased risk of hypertension in older adults. These results were consistent for divorced or widowed individuals who transitioned from living with family to living alone. The present study emphasizes the significance of social support and connectedness in maintaining cardiovascular health among older adults. Health care providers and policymakers may need to consider interventions that focus on increasing social support and reducing social isolation to prevent hypertension among the older population.

The number of older adults living alone has become an important social issue in today’s aging society (1). A dramatic increase in the proportion of older adults living alone has been observed over the years. In China, 9.7% of older adults lived alone in 2010 (2), with the rate increasing to 23.3% in rural regions in 2015 (3). As of 2018, the number of people living alone in China has reached 240 million, increasing by 0.53% annually (4). Factors such as the decreasing demand between parents and children, sufficient family resources, the increase in the homeownership rate, the greater autonomy of widowed older adults, and the extended period of self-care may contribute to this trend (5).

However, an increasing body of evidence suggests that older adults living alone are more likely than those living with their families to experience feelings of loneliness, and isolation (6,7), which could exacerbate primary illnesses and negatively affect their quality of life. Living alone has numerous negative health consequences, including myocardial infarction (8), diabetes mellitus (9), dementia (10), depression (11), and death (12). It is widely acknowledged that hypertension is one of the most common chronic diseases affecting more than half of the population older than 65 years and nearly 90% of those older than 80 years in China (13). Although several population-based studies have focused on the relationship between living alone and hypertension, their findings have been largely inconsistent. For example, two cross-sectional studies investigated the relationship between living alone and the risk of hypertension in adults aged 45 years or older (14,15). One study found that living alone was linked to lower hypertension risk in women (14), while the other study found the same association in men (15). Conversely, one cross-sectional study reported that older adults who lived alone had higher odds of hypertension than those who lived with others (16), while another showed that living alone was not associated with hypertension (17). Nonetheless, it should be borne in mind that cross-sectional studies have limitations in establishing causality. Little is currently known about whether the results of previous studies can be generalized to older adults, nor is it clear whether there is an association between changes in living arrangements and hypertension. Given the increasing number of older adults living alone and the high prevalence of hypertension, prospective studies are needed to clarify this critical issue.

Therefore, this study aimed to examine the association between living alone, transitions in living arrangements, and hypertension in older adults using data from the Chinese Longitudinal Healthy Longevity Survey (CLHLS).

## Method

### Study Design and Participants

The CLHLS is an extensive ongoing prospective cohort study that is being conducted in half of the counties or municipalities in 22 out of China’s 31 provinces. This national cohort focuses primarily on older adults and contains individual-level information on demographic and health indicators, socioeconomic characteristics, and social and behavioral risk factors. The quality of the data collected for this study was considered high based on systematic assessments of attrition randomness, credibility and validity of the measurement scale, and accuracy of reported age (18). The baseline wave was conducted in 1998, followed by face-to-face follow-up surveys every 2 to 4 years. Detailed descriptions of the CLHLS have been published elsewhere (18).

This study included CLHLS data from 2008 (fifth wave) and 2011/2012 (sixth wave) due to the absence of the results of two blood pressure measurements in the previous waves. We merged the data of these two waves into one data set as baseline data. The 2008 wave and 2011/2012 wave included 26 719 older Chinese individuals. A follow-up of the 2008 wave was conducted in 2011/2012 and a follow-up of the 2011/2012 wave in 2014. To analyze the association between living arrangements and hypertension in older adults, we excluded participants based on the following reasons: older adults aged <65 years, lost to follow-up or uncertain follow-up dates, missing data on blood pressure and living arrangements, participants with physician-diagnosed hypertension or systolic blood pressure (SBP) ≥140 mmHg and/or diastolic blood pressure (DBP) ≥90 mmHg, duplicate participants across the two waves, and residing in a nursing home. Finally, a total of 8 782 participants without hypertension at baseline were included in a cohort. To analyze the association between living arrangement transitions and hypertension, we excluded those who died during follow-up, those with missing data on living arrangements in the follow-up wave, and those living in nursing homes in the follow-up wave. Supplementary Figure 1 shows the detailed process for the inclusion and exclusion of study participants. All participants or their relatives gave their informed consent. The Biomedical Ethics Committee of Peking University approved this study (IRB00001052-13074).

### Assessment of Living Arrangements

The living arrangements of the participants were assessed using a questionnaire that asked who they were living with. Living arrangements were documented as either living alone or living with family. We compared living alone with the risk of hypertension.

To examine changes in living arrangements during follow-up, we identified four categories: (1) Not alone/Not alone; (2) Not alone/Alone; (3) Alone/Not alone; and (4) Alone/Alone. Marital status in the follow-up wave was examined to identify factors associated with change. Using additional measures concerning marital status, six categories were used to describe the type of living arrangements: (1) Not alone/Not alone (married); (2) Not alone/Not alone (widowed/divorced); (3) Not alone/Alone (married); (4) Not alone/Alone (widowed/divorced); (5) Alone/Not alone; and (6) Alone/Alone. As the number of participants was limited, it was not feasible to categorize those who lived alone or switched from living alone to living with family based on their marital status.

### Outcome Assessment

Participants who developed hypertension during the ­follow-up and those with hypertension before death were included in the outcome group with hypertension. Regardless of whether they were alive or dead, the remaining participants were included in the outcome group without hypertension.

The blood pressure of participants was measured twice in their right arm by trained investigators using a mercury sphygmomanometer (upper arm type; Yuyue, Jiangsu, China). Prior to the measurement, the participants were instructed to rest for a minimum of 5 minutes. The interval between the two measurements was not less than 1 minute, and the average value was used for all further analyses. For survivor respondents, hypertension was defined as SBP ≥140 mmHg and/or DBP ≥90 mmHg or a self-reported history of hypertension diagnosed by a physician (13). For deceased respondents, hypertension status was determined from close relatives based on the question, “Was the participant suffering from hypertension before passing away?.”

### Assessment of Covariates

We controlled for potential confounding variables associated with living alone or hypertension in our analyses: age (continuous), gender, education (no school or 1 year or more), residence (urban or rural), marital status (married vs other (widowed, divorced, or never married), smoking (never, current, or former), drinking (never, current, or former), regular exercise (never, current, or former), activities of daily living (ADL) limitations (yes or no), annual household income (<10 000, 10 000–30 000, or >30 000 yuan), sleep time (<6, 6–9, or ≥9 h), body mass index (BMI) and chronic complications included self-reported diabetes (yes or no), stroke or cardiovascular disease (CVD) (yes or no), respiratory disease (including bronchitis, emphysema, and pneumonia) (yes or no), and cancer (yes or no).

For the current study, ADL was assessed using the Katz index scale, which evaluates an individual’s ability to carry out essential daily tasks such as bathing, dressing, toileting, indoor transferring, continence, and feeding (19). ADL limitation is defined as difficulty or a need for assistance in one or more tasks (20). To obtain height and weight measurements, trained staff followed standard operating procedures. Height was assessed using a portable stadiometer while participants stood barefoot. Weight was measured on a scale with participants wearing light indoor clothing and no shoes. BMI was calculated as the quotient of weight (in kg) and the square of height (in m). We classified BMI into underweight (<18.5 kg/m^2^), normal (18.5–24 kg/m^2^), overweight (24–28 kg/m^2^), and obese (≥28 kg/m^2^) (21).

### Statistical Analysis

We adopted a multiple imputation method based on the chained equation approach and the five repetitions method to deal with the missing data (ranging from 0.07% to 1.80%; Supplementary Table 1). The data were presented as medians and interquartile ranges (IQRs) for continuous variables and percentages for categorical variables.

The crude incidence rate (IR) (per 100 person-years) of hypertension across categories of living arrangements was estimated. Cox proportional hazards models were used to evaluate the association between living arrangements and hypertension. To analyze all outcomes in the study, three model types were constructed: an unadjusted model, a model adjusted for several covariates such as age, gender, educational level, residence, marital status, annual household income, drinking status, smoking status, regular exercise, and ADL limitation (model 1), and a model further adjusted for additional factors, including sleep time, BMI, diabetes, heart disease, stroke, CVD, respiratory disease, and cancer (model 2). Additionally, a separate analysis was conducted to examine the relationship between changes in living arrangements during follow-up and hypertension risk. The results of the study were presented as combined hazard ratios (HRs) with 95% confidence intervals (95% CIs).

Stratified and interaction analyses were conducted according to living arrangements and participant characteristics (age, gender, and residence). We also conducted a series of sensitivity analyses to assess the robustness of our findings. First, we constructed a competing risk model to evaluate the relationship between living arrangements and hypertension, whereby death without hypertension was considered a competitive event. Second, we performed propensity score matching (PSM) to eliminate confounding factors by balancing baseline covariates between the observation (living alone) and control (living with family) groups and eliminate potential confounding variables, simulating a randomized controlled trial. This was done to account for possible biases and ensure the accuracy of our results (22). Propensity score matching was conducted without replacement in a 1:1 ratio with caliper widths equal to 0.05 using the nearest-neighbor matching algorithm. We used standardized mean difference (SMD) to evaluate the intergroup balance. An SMD of less than 0.1 is considered to indicate a balance between the groups (23). Third, we excluded participants who were dead during follow-up to mitigate potential confounding effects. Fourth, we excluded participants who suffered from hypertension in the first 1 year of follow-up to mitigate and eliminate the potential influence of short-term follow-up because hypertension is typically a chronic condition that develops gradually over time. Finally, we excluded participants with diabetes, heart disease, stroke, CVD, respiratory disease, or cancer at baseline to avoid potential confounding effects from comorbidities.

All analyses were performed using R statistical software version 4.1.3 (R Foundation for Statistical Computing). A two-tailed *p* value less than .05 was set as the significance threshold for all analyses.

## Results

### Basic Characteristics of Participants

As shown in Table 1, participants who lived alone (15.4%) tended to be younger, unmarried (divorced, widowed, or unmarried) and lived in rural areas compared to those who lived with the family. They were also more likely to be nonsmokers and not engage in regular exercise, have no ADL limitations, report lower annual household income, short sleep time, and have a lower proportion of stroke or CVD.

**Table 1.** Demographic and Clinical Characteristics of the Study Population

Characteristics	Overall (*n* = 8 782)	Living With Family (*n* = 7 433)	Living Alone (*n* = 1 349)	*p* Value
Age (years), median (IQR)	89.00 (78.00, 97.00)	89.00 (78.00, 97.00)	87.00 (79.00, 94.00)	<.001
Female, *n* (%)	4 894 (55.7)	4 113 (55.3)	781(57.9)	.087
Married, *n* (%)	2 952 (33.6)	2 878 (38.7)	74(5.5)	<.001
Urban area, *n* (%)	3 318 (37.8)	2 865 (38.5)	453(33.6)	.001
Education (year), *n* (%)				<.001
0	5 532 (63.0)	4 609 (62.0)	923(68.4)	
≥1	3 250 (37.0)	2 824 (38.0)	426(31.6)	
Smoking status, *n* (%)				.032
Never	5 813 (66.2)	4 890 (65.8)	923(68.4)	
Current	1 625 (18.5)	1 374 (18.5)	251(18.6)	
Former	1 344 (15.3)	1 169 (15.7)	175(13.0)	
Drinking status, *n* (%)				.861
Never	5 950 (67.8)	5 033 (67.7)	917(68.0)	
Current	1 638 (18.7)	1 393 (18.7)	245(18.2)	
Former	1 194 (13.6)	1 007 (13.5)	187(13.9)	
Regular exercise, *n* (%)				<.001
Never	5 389 (61.4)	4 523 (60.9)	866(64.2)	
Current	2 340 (26.6)	1 976 (26.6)	364(27.0)	
Former	1 053 (12.0)	934 (12.6)	119(8.8)	
ADL limitation	1 978 (22.5)	1 851 (24.9)	127(9.4)	<.001
Annual household income (yuan), *n* (%)				<.001
<10 000	3 604 (41.0)	2 661 (35.8)	943(69.9)	
10 001-30 000	2 954 (33.6)	2 710 (36.5)	244(18.1)	
>30 000	2 224 (25.3)	2 062 (27.7)	162(12.0)	
Sleep time (h), *n* (%)				.001
<6	1 041 (11.9)	840 (11.3)	201(14.9)	
6–9	4 491 (51.1)	3 820 (51.4)	671(49.7)	
≥9	3 250 (37.0)	2 773 (37.3)	477(35.4)	
BMI (kg/m^2^), *n* (%)				.794
Underweight (<18.5)	3 116 (35.5)	2 645 (35.6)	471(34.9)	
Normal (18.5–24)	4 655 (53.0)	3 927 (52.8)	728(54.0)	
Overweight (24–28)	808 (9.2)	691 (9.3)	117(8.7)	
Obese (≥28)	203 (2.3)	170 (2.3)	33(2.4)	
Diabetes, *n* (%)	144 (1.6)	122 (1.6)	22(1.6)	1.000
Heart disease, *n* (%)	555(6.3)	473(6.4)	82(6.1)	.738
Stroke or CVD, *n* (%)	411 (4.7)	367(4.9)	44(3.3)	.009
Respiratory disease, *n* (%)	964 (11.0)	822 (11.1)	142(10.5)	.597
Cancer, *n* (%)	40 (0.5)	34 (0.5)	6(0.4)	1.000

*Note*: ADL = activities of daily living; BMI = body mass index; CVD = cardiovascular diseases; IQR = interquartile range.

After performing the PSM, 2 360 participants were included in the analysis. Standardized mean differences of the covariates were less than 0.1, indicating that the resultant groups from PSM were well-balanced and had no significant confounding effects (Supplementary Figure 2). The baseline variables were successfully balanced between living alone and living with family groups after PSM (Supplementary Table 2).

### Association of Living Arrangements with Hypertension

The study included 8 782 participants who were followed up for a total of 20 110.3 person-years (median follow-up of 2.8 years). During the follow-up period, 2 750 participants (31.3%) developed hypertension, of whom 2 263 lived with family and 487 lived alone. Among these, 1 310 (47.6%) were diagnosed based solely on actual blood pressure measurements, 753 (27.4%) were diagnosed based on self-reported medical history of hypertension diagnosed by a physician, and 687 (25.0%) were diagnosed using both methods. The IR for hypertension was higher among participants who lived alone than those who lived with family (IR per 100 person-years: 15.31 vs 13.37; Table 2). Living alone was associated with an increased risk of hypertension (HR = 1.19; 95% CI: 1.06–1.33) in the fully adjusted model (Table 2).

**Table 2.** Hypertension Risk According to Living Arrangements

Variables	No. of events (n)/Incidence rate (per 100 person-years, %)	Unadjusted Model		Model 1^*^		Model 2^†^	
		HR (95% CI)	*p* Value	HR (95% CI)	*p* Value	HR (95% CI)	*p* Value
Living with family	2 263 (13.37)	Reference		Reference		Reference	
Living alone	487 (15.31)	1.12 (1.02–1.24)	.019	1.18 (1.06–1.32)	.003	1.19 (1.06–1.33)	.003

*Notes*: CI = confidence interval; HR = hazard ratio.

^*^Model 1 was adjusted for age, gender, educational level, residence, marital status, annual household income, drinking status, smoking status, regular exercise, and activities of daily living limitation.

^†^Model 2 was further adjusted for sleep time, body mass index, diabetes, heart disease, stroke, cardiovascular diseases, respiratory disease, and cancer.

To ensure the reliability of our study’s findings, we conducted a series of sensitivity analyses. The competing risk model and PSM results were consistent with our primary analysis. We found that this association remained robust even after we excluded participants who had hypertension in the first year of follow-up. Furthermore, our fully adjusted model, which only included participants with complete data or excluded those who were deceased during the follow-up period, or those with comorbidities such as diabetes, heart disease, stroke, CVD, respiratory disease, or cancer, showed consistency with our initial adjusted and imputed model (Supplementary Table 3).

### Association of Living Arrangements With Hypertension by Subgroup

**Figure 1.**
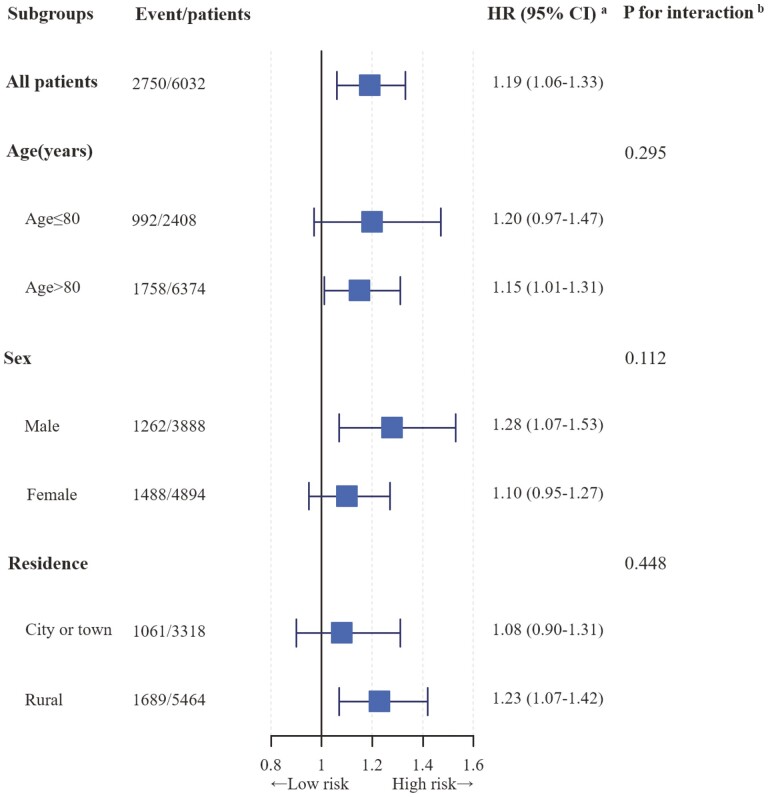
Association of living arrangements with hypertension stratified by participant characteristics. *Notes*: HR = hazard ratio; CI = confidence interval; CVD = cardiovascular diseases. ^a^Each stratification controlled for all factors (age, gender, educational level, residence, marital status, annual household income, drinking status, smoking status, regular exercise, activities of daily living limitation, sleep time, body mass index, diabetes, heart disease, stroke, cardiovascular diseases, respiratory disease, and cancer) except the stratification factor itself. ^b^Test for the interaction between living status (living with family and living alone) and participant characteristics.

We performed a stratified analysis based on participant characteristics, including age, gender, and residence. Our findings indicated that living alone was associated with a 20% and 28% higher risk of hypertension in older adults aged more than 80 and males, respectively. However, we found no significant association in females or older adults aged 80 or younger. Notably, older adults living alone in rural areas had a 23% higher risk of hypertension, while no such association was observed in those residing in cities or towns (Figure 1). We found no significant interactions between living arrangements and participant characteristics (*p*-interaction >.05 for all; Figure 1).

### Association of Hypertension and Transitions in Living Arrangements

Supplementary Table 4 shows that participants who had continued to live alone had a higher risk of hypertension than those who continued to live with family (HR = 1.24; 95% CI: 1.06–1.45) in the final model.

Table 3 shows the association between hypertension and living arrangement transitions stratified by marital status. In the fully adjusted model, compared with married participants who continuously lived with family, participants who had always lived alone, and widowed/divorced participants who reported switching from living with family to living alone had a significantly higher risk of hypertension, with HRs of 1.35 (95% CI: 1.10–1.66) and 1.21 (95% CI: 1.00–1.47), respectively. In contrast, married participants who previously lived with family and then lived alone did not show a higher risk of hypertension. Similarly, there was no additional risk for participants transitioning from living alone to living with family.

**Table 3.** Association Between Hypertension and Living Arrangement Transitions Stratified by Marital Status

Variables	Unadjusted Model		Model 1^*^		Model 2^†^	
	HR (95% CI)	*p* Value	HR (95% CI)	*p* Value	HR (95% CI)	*p* Value
Not alone/not alone (married) (*n* = 1 872)	Reference		Reference		Reference	
Not alone/not Alone (widowed/divorced) (*n* = 1 997)	1.10 (1.00–1.21)	.050	1.09 (0.93–1.28)	.265	1.10 (0.94–1.29)	.241
Not alone/alone (married) (*n* = 82)	0.87 (0.62–1.22)	.417	0.88 (0.63–1.24)	.472	0.85 (0.61–1.20)	.361
Not alone/alone (widowed/divorced) (*n* = 324)	1.24 (1.04–1.47)	.016	1.22 (1.01–1.48)	.041	1.21 (1.00–1.47)	.049
Alone/not alone (*n* = 354)	1.14 (0.97–1.35)	.115	1.20 (0.97–1.49)	.091	1.21 (0.98–1.50)	.075
Alone/alone (*n* = 497)	1.24 (1.07–1.44)	.004	1.35 (1.10–1.66)	.004	1.35 (1.10–1.66)	.004

*Notes*: CI = confidence interval; HR = hazard ratio.

^*^Model 1 was adjusted for age, gender, educational level, residence, marital status at baseline, annual household income, drinking status, smoking status, regular exercise, and activities of daily living limitation.

^†^Model 2 was further adjusted for sleep time, body mass index, diabetes, heart disease, stroke, cardiovascular diseases, respiratory disease, and cancer.

## Discussion

This prospective cohort study examined the association between living alone, changes in living arrangements, and hypertension among older adults. We found that older adults who reported living alone at baseline and persistently living alone during the follow-up had a significantly increased risk of hypertension. In addition, widowed/divorced participants who reported transitioning from living with family to living alone during the follow-up period had a significantly higher risk of hypertension than married participants who remained living with family. Our findings contribute to the existing literature by providing further evidence of the association between living arrangements and hypertension.

Moreover, we found a positive correlation between living alone and the risk of hypertension, consistent with previous cross-sectional studies (16,24). Redondo-Sendino et al. reported that the risk of hypertension was lower in married individuals and those living with others than in unmarried individuals or those living alone, based on a study of 3 483 Spanish individuals aged 60 years or older (24). Notably, our study overcame the limitations of cross-sectional designs in establishing causal links, thereby providing robust evidence on the relationship between living alone and hypertension risk. In contrast with our findings, two cross-sectional studies (14,15) showed that living alone was associated with a lower risk of hypertension than living with others among middle-aged and older adults (14,15). Plausible rationales for observed variations in hypertension risk among study participants may stem from geographical location, ethnicity, age, or lifestyle disparities. Distinct from prior research limited to specific geographic regions, our investigation covers a broad scope of 23 research sites across mainland China. Furthermore, since our study population comprised older Chinese adults with advanced age, there may be variations in dietary patterns, physical activity levels, and living habits that could lead to different associations between living alone and hypertension. To minimize the impact of reverse causation bias, we employed additional measures by eliminating participants with preexisting chronic illnesses including diabetes, heart disease, stroke, CVD, respiratory disease, or cancer, and also those who developed hypertension within the first year of follow-up. This approach proved effective, as the results indicated no significant alterations. Despite our efforts, completely eliminating reverse causation may not be possible. We further conducted a subgroup analysis to explore potential effect modifiers. Our findings indicated that living alone was associated with increased hypertension risk among participants older than 80 years, men, and rural residents.

Though the association between living alone and hypertension risk remains in question, some potential explanations exist for gender, age, and regional differences. One possible explanation for these gender, age, and regional differences could be attributed to the social control hypothesis. This theory emphasizes the role of family in shaping a person’s health behaviors. Older adults who live alone may lack the social support and influence that comes with living with family members and may be more exposed to cardiovascular risk factors like physical inactivity, substance abuse, reduced sleep, limited dietary diversity, less frequent consumption of vegetables, fruits, and seafood, and increased intake of salty foods, particularly among men (25,26). Besides, people who live with their families may have better access to medical services when they become ill, enabling early intervention for risk factors. In this respect, we found a strong association between living alone and hypertension among older adults in rural areas due to several factors, including poor infrastructure, limited economic resources, and limited access to health information, all of which may prevent these individuals from engaging in health-promoting activities (27), resulting in higher stress levels than older people living alone in urban areas. Living alone may also lead to social isolation and loneliness due to reduced social networking and increased feelings of social disconnection. This can negatively affect one’s physical and mental health, including an increased risk of hypertension (11,28). In addition, social isolation and loneliness can lead to the activation of the hypothalamic–pituitary–adrenal axis (29), resulting in the increased secretion of glucocorticoids (30). It is widely acknowledged that glucocorticoids contribute to elevated SBP and the development of atherosclerosis by increasing vasoconstriction (31), reducing endothelial nitric oxide production (32), and enhancing oxidative stress (33). Overwhelming evidence substantiates that lonely people have greater total peripheral vascular resistance (34,35), accounting for a higher risk of hypertension (36). Current evidence suggests that men who live alone develop isolation and depression significantly earlier than women (37), possibly because men tend to have fewer social networks than women (38). Herein, we also observed that the association between living alone and hypertension was stronger in people older than 80, suggesting that age changes the association between living alone and hypertension. One potential explanation for this modification is that for some younger older adults, living alone may be a choice made to pursue a free or private lifestyle (39), and as a result, they may be less likely to experience depression, isolation, or loneliness than older adults who live alone. Furthermore, studies have reported an association between living alone and higher levels of interleukin 6 and/or C-reactive protein (40,41). Systemic inflammation has been established to play a key role in the pathophysiology of CVDs, such as hypertension (42).

According to a previous study, older adults who began living alone due to divorce or widowhood were at a higher risk of mortality. Contrastingly, individuals who previously lived with someone other than their spouse and then transitioned to living alone did not exhibit the same increased risk (43). This suggests that older adults who lose the support of their closest relationships are at greater risk of death. However, there are no studies on the effect of changes in living arrangements on the development of hypertension. Our study found that those who lived alone had an increased risk of hypertension compared to those who continued living with family. After stratification according to marital status and changes in living arrangements at follow-up, we found that widowed or divorced participants had an increased risk of hypertension after moving from living with family to living alone, but not for married participants who had previously lived with family but later lived alone. This suggests that divorced or widowed older adults will face a greater risk of hypertension when they live alone, even after considering factors such as age, gender, and socioeconomic status. This is consistent with evidence that being widowed led to an increased risk of hypertension (44,45). As people age, some may find themselves living alone due to the passing of a spouse, leaving them with no other viable option. For these individuals, living alone may become a permanent arrangement. However, some older adults choose to live alone during their marriage for a variety of reasons. For instance, they may need to care for their children or grandchildren or work outside the home. Although couples live apart, the emotional support and social controls against unhealthy lifestyles (such as smoking, alcohol consumption, physical inactivity, etc.) that marriage provides may remain (46). This support system can help individuals to avoid unhealthy habits such as smoking, excessive drinking, or physical inactivity, potentially reducing their risk of hypertension. Additionally, couples living apart can maintain strong social connections with their families and build their support network, mitigating potential health risks associated with living alone.

Living alone is often used as a measure or indicator of social isolation (47). Our study suggests that living alone is an easily recognized risk factor for hypertension, which has important implications for clinical practice. Emphasis should be placed on the risk of increased blood pressure and the resulting adverse health risks to older adults when they transition from living with family to living alone, especially for older adults who are widowed or divorced. Our findings hold potential significance for developing intervention strategies for older adults living alone, such as implementing social prescriptions (www.kingsfund.org.uk/publications/social-prescribing) to control the rapid increase in hypertension prevalence among older adults living alone in China.

### Strengths and Limitations

Herein, we analyzed a large sample of community-dwelling older adults in China to investigate the link between living alone and hypertension. We also assessed how the risk of hypertension changed during the transition between living arrangements. However, this study still has several limitations. First, all included participants were older adults from China, and our findings may not apply to other populations and younger people. Second, most covariate information was collected by questionnaire, and comorbidities were self-reported. Therefore, recall bias may not be avoidable. Third, the participants’ employment status and diet were unknown, which may have influenced the results. Fourth, 75.0% of the participants were diagnosed with hypertension based on self-reported or actual blood pressure measurements alone in our study. Indeed, relying solely on self-reported hypertension can introduce bias into our findings, and measuring blood pressure in a research setting may not always reflect an individual’s true blood pressure in their daily life, as some individuals may experience “white coat hypertension” or elevated blood pressure in a clinical setting. Future studies could employ a combination of self-report and actual blood pressure measurements to gain a more comprehensive understanding of hypertension status. Finally, the exact timing of the change in living arrangements was not investigated during the follow-up. Accordingly, we could not confirm the effect of time spent alone on hypertension.

## Conclusion

Our findings suggest that living alone is associated with a higher risk of hypertension and that divorced or widowed older adults who transitioned from living with their families to living alone are also at increased risk. This study found that the relationship between living arrangements and hypertension among older adults is influenced by age, gender, and residence. Our results suggest that interventions focused on increasing social support and decreasing social isolation may have positive effects on controlling blood pressure in this demographic. Nonetheless, further studies are warranted, including a more objective assessment of the impact of time spent alone on hypertension risk, to confirm our findings.

## Supplementary Material

igad071_suppl_Supplementary_Maretial

## References

[1] Rico-Uribe LA , Caballero FF, Martín-María N, Cabello M, Ayuso-Mateos JL, Miret M. Association of loneliness with all-cause mortality: a meta-analysis. PLoS One. 2018;13(1):e0190033. doi:10.1371/journal.pone.0190033 29300743 PMC5754055

[2] China Scientific Research Center on Aging. Summary of Main Data of China’s Urban and Rural Elderly Population Tracking Survey. (2010). 2021. http://www.crca.cn/index.php/19-life/26-2010.html.

[3] Liu H , Zhuang Y, Liang Y, et al. China family development report (2015). China Popul Dev Stud. 2017;1:98–115. doi:10.1007/bf03500920

[4] Long H , Shi S, Tang Z, Zhang S. Does living alone increase the consumption of social resources? Environ Sci Pollut Res Int. 2022;29(47):71911–71922. doi:10.1007/s11356-022-20892-w 35610449

[5] Li M , Zhang L, Xiong W. Analysis on the living style and influencing factors of the widowed elderly in China-based on data from the fourth sample survey of the living conditions of China’s urban and rural elderly in 2015. Res World. 2019;2:24–28.

[6] Lim LL , Kua EH. Living alone, loneliness, and psychological well-being of older persons in singapore. Curr Gerontol Geriatr Res. 2011;2011:673181. doi:10.1155/2011/673181 21969827 PMC3182578

[7] Iamtrakul P , Chayphong S. Exploring the influencing factors on living alone and social isolation among older adults in rural areas of Thailand. Int J Environ Res Public Health. 2022;19(21):14572. doi:10.3390/ijerph192114572 36361450 PMC9655045

[8] Kilpi F , Konttinen H, Silventoinen K, Martikainen P. Living arrangements as determinants of myocardial infarction incidence and survival: a prospective register study of over 300,000 Finnish men and women. Soc Sci Med. 2015;133:93–100. doi:10.1016/j.socscimed.2015.03.054 25863724

[9] Meisinger C , Kandler U, Ladwig KH. Living alone is associated with an increased risk of type 2 diabetes mellitus in men but not women from the general population: the MONICA/KORA Augsburg Cohort Study. Psychosom Med. 2009;71(7):784–788. doi:10.1097/PSY.0b013e3181ae5770 19592514

[10] Shaw BA , Yang TC, Kim S. Living alone during old age and the risk of dementia: assessing the cumulative risk of living alone. J Gerontol Series B Psychol Sci Soc Sci. 2022;78(2):293–301. doi:10.1093/geronb/gbac156 PMC9938918 36179214

[11] Lim YM , Baek J, Lee S, Kim JS. Association between loneliness and depression among community-dwelling older women living alone in South Korea: the mediating effects of subjective physical health, resilience, and social support. Int J Environ Res Public Health. 2022;19(15):9246. doi:10.3390/ijerph19159246 35954597 PMC9368532

[12] Zhao Y , Guyatt G, Gao Y, et al. Living alone and all-cause mortality in community-dwelling adults: a systematic review and meta-analysis. EClinicalMedicine. 2022;54:101677. doi:10.1016/j.eclinm.2022.101677 36204005 PMC9530481

[13] Hua Q , Fan L, Li J. 2019 Chinese guideline for the management of hypertension in the elderly. J Geriatr Cardiol. 2019;16(2):67–99. doi:10.11909/j.issn.1671-5411.2019.02.001 30923539 PMC6431598

[14] Kim YJ. Association of family composition and metabolic syndrome in Korean adults aged over 45 years old. Asian Nurs Res. 2015;9(4):349–355. doi:10.1016/j.anr.2015.10.006 26724245

[15] Hosseini Z , Veenstra G, Khan NA, Conklin AI. Social connections and hypertension in women and men: a population-based cross-sectional study of the Canadian Longitudinal Study on Aging. J Hypertens. 2021;39(4):651–660. doi:10.1097/HJH.0000000000002688 33065735

[16] Tan S , Liu D, Zhang Y, Li S, Zhang K, Zuo H. Trends in blood pressure and hypertension among older adults and oldest-old individuals in China between 2008-2018. Hypertens Res. 2023;46(5):1145–1156. doi:10.1038/s41440-023-01183-4 36750610

[17] Davarian S , Crimmins E, Takahashi A, Saito Y. Sociodemographic correlates of four indices of blood pressure and hypertension among older persons in Japan. Gerontology. 2013;59(5):392–400. doi:10.1159/000350531 23689609 PMC3844551

[18] Zeng Y , Feng Q, Hesketh T, Christensen K, Vaupel JW. Survival, disabilities in activities of daily living, and physical and cognitive functioning among the oldest-old in China: a cohort study. Lancet. 2017;389(10079):1619–1629. doi:10.1016/S0140-6736(17)30548-2 28285816 PMC5406246

[19] Katz S , Ford AB, Moskowitz RW, Jackson BA, Jaffe MW. Studies of illness in the aged. The index of ADL: a standardized measure of biological and psychosocial function. JAMA. 1963;185:914–919. doi:10.1001/jama.1963.03060120024016 14044222

[20] Fuller-Thomson E , Yu B, Nuru-Jeter A, Guralnik JM, Minkler M. Basic ADL disability and functional limitation rates among older Americans from 2000-2005: the end of the decline? J Gerontol A Biol Sci Med Sci. 2009;64(12):1333–1336. doi:10.1093/gerona/glp130 19723771 PMC2781786

[21] Wang J , TaylorAW, ZhangT, AppletonS, ShiZ. Association between body mass index and all-cause mortality among oldest old Chinese. J Nutr Health Aging. 2018;22(2):262–268. doi:10.1007/s12603-017-0907-229380854

[22] Deb S , Austin PC, Tu JV, et al. A review of propensity-score methods and their use in cardiovascular research. Can J Cardiol. 2016;32(2):259–265. doi:10.1016/j.cjca.2015.05.015 26315351

[23] Benedetto U , Head SJ, Angelini GD, Blackstone EH. Statistical primer: propensity score matching and its alternatives. Eur J Cardiothorac Surg. 2018;53(6):1112–1117. doi:10.1093/ejcts/ezy167 29684154

[24] Redondo-Sendino A , Guallar-Castillón P, Banegas JR, Rodríguez-Artalejo F. Relationship between social network and hypertension in older people in Spain. Rev Esp Cardiol. 2005;58(11):1294–1301. 16324583

[25] Jeong S , Cho SI. Effects of living alone versus with others and of housemate type on smoking, drinking, dietary habits, and physical activity among elderly people. Epidemiol Health. 2017;39:e2017034. doi:10.4178/epih.e2017034 29121710 PMC5675988

[26] Nakashima T , Katayama N, Saji N, et al. Dietary habits and medical examination findings in Japanese adults middle-aged or older who live alone. Nutrition. 2021;89:111268. doi:10.1016/j.nut.2021.111268 34091192

[27] Oh H , Noh H, Sims OT, Guo Y, Sawyer P. A comparison of urban and non-urban African American older adults on health-related characteristics. Soc Work Health Care. 2018;57(9):762–773. doi:10.1080/00981389.2018.1497748 30118652

[28] Russell D , Taylor J. Living alone and depressive symptoms: the influence of gender, physical disability, and social support among Hispanic and non-Hispanic older adults. J Gerontol B Psychol Sci Soc Sci. 2009;64(1):95–104. doi:10.1093/geronb/gbn002 19176487 PMC2654980

[29] Cacioppo JT , Cacioppo S, Capitanio JP, Cole SW. The neuroendocrinology of social isolation. Annu Rev Psychol. 2015;66:733–767. doi:10.1146/annurev-psych-010814-015240 25148851 PMC5130104

[30] Adam EK , Hawkley LC, Kudielka BM, Cacioppo JT. Day-to-day dynamics of experience—cortisol associations in a population-based sample of older adults. Proc Natl Acad Sci U S A. 2006;103(45):17058–17063. doi:10.1073/pnas.0605053103 17075058 PMC1636578

[31] Burford NG , Webster NA, Cruz-Topete D. Hypothalamic-pituitary-adrenal axis modulation of glucocorticoids in the cardiovascular system. Int J Mol Sci. 2017;18(10):2150. doi:10.3390/ijms18102150 29035323 PMC5666832

[32] Wallerath T , Witte K, Schäfer SC, et al. Down-regulation of the expression of endothelial NO synthase is likely to contribute to glucocorticoid-mediated hypertension. Proc Natl Acad Sci U S A. 1999;96(23):13357–13362. doi:10.1073/pnas.96.23.13357 10557325 PMC23952

[33] Colaianna M , Schiavone S, Zotti M, et al. Neuroendocrine profile in a rat model of psychosocial stress: relation to oxidative stress. Antioxid Redox Signal. 2013;18(12):1385–1399. doi:10.1089/ars.2012.4569 23320850 PMC3603501

[34] Cacioppo JT , Hawkley LC, Crawford LE, et al. Loneliness and health: potential mechanisms. Psychosom Med. 2002;64(3):407–417. doi:10.1097/00006842-200205000-00005 12021415

[35] Hawkley LC , Burleson MH, Berntson GG, Cacioppo JT. Loneliness in everyday life: cardiovascular activity, psychosocial context, and health behaviors. J Pers Soc Psychol. 2003;85(1):105–120. doi:10.1037/0022-3514.85.1.105 12872887

[36] Hawkley LC , Thisted RA, Masi CM, Cacioppo JT. Loneliness predicts increased blood pressure: 5-year cross-lagged analyses in middle-aged and older adults. Psychol Aging. 2010;25(1):132–141. doi:10.1037/a0017805 20230134 PMC2841310

[37] Koo JH , Son N, Yoo KB. Relationship between the living-alone period and depressive symptoms among the elderly. Arch Gerontol Geriatr. 2021;94:104341. doi:10.1016/j.archger.2021.104341 33497913

[38] Ajrouch KJ , Blandon AY, Antonucci TC. Social networks among men and women: the effects of age and socioeconomic status. J Gerontol B Psychol Sci Soc Sci. 2005;60(6):S311–S317. doi:10.1093/geronb/60.6.s311 16260713

[39] de Jong Gierveld J , Dykstra P, Schenk N. Living arrangements, intergenerational support types and older adults loneliness in Eastern and Western Europe. Demogr Res. 2012;27:167–200.

[40] Walker E , Ploubidis G, Fancourt D. Social engagement and loneliness are differentially associated with neuro-immune markers in older age: time-varying associations from the English Longitudinal Study of Ageing. Brain Behav Immun. 2019;82:224–229. doi:10.1016/j.bbi.2019.08.189 31491488 PMC6997881

[41] Zilioli S , Jiang Y. Endocrine and immunomodulatory effects of social isolation and loneliness across adulthood. Psychoneuroendocrinology. 2021;128:105194. doi:10.1016/j.psyneuen.2021.105194 33932766

[42] Oparil S , Zaman MA, Calhoun DA. Pathogenesis of hypertension. Ann Intern Med. 2003;139(9):761–776. doi:10.7326/0003-4819-139-9-200311040-00011 14597461

[43] Abell JG , Steptoe A. Why is living alone in older age related to increased mortality risk? A longitudinal cohort study. Age Ageing. 2021;50(6):2019–2024. doi:10.1093/ageing/afab155 34304269 PMC8675439

[44] Perkins JM , Lee HY, James KS, et al. Marital status, widowhood duration, gender and health outcomes: a cross-sectional study among older adults in India. BMC Public Health. 2016;16(1):1032. doi:10.1186/s12889-016-3682-9 27716203 PMC5045657

[45] Pantell MS , Prather AA, Downing JM, Gordon NP, Adler NE. Association of social and behavioral risk factors with earlier onset of adult hypertension and diabetes. JAMA Netw Open. 2019;2(5):e193933. doi:10.1001/jamanetworkopen.2019.3933 31099868 PMC6537925

[46] Umberson D. Gender, marital status and the social control of health behavior. Soc Sci Med. 1992;34(8):907–917. doi:10.1016/0277-9536(92)90259-s 1604380

[47] Desai R , John A, Stott J, Charlesworth G. Living alone and risk of dementia: a systematic review and meta-analysis. Ageing Res Rev. 2020;62:101122. doi:10.1016/j.arr.2020.101122 32659336

