# Supplementary material for: Living Alone Increases the Risk of Hypertension in Older Chinese Adults: A Population-Based Longitudinal Study
Source: Innov Aging. 2023 Jul 3;7(6):igad071. doi: 10.1093/geroni/igad071 (PMC10370894; doi:10.1093/geroni/igad071)
Supplement: igad071_suppl_Supplementary_Maretial [file igad071_suppl_supplementary_maretial.docx]

**Online Supplementary Material**

- **Supplementary Table 1.** Distributions of variables with missing data comparing observed complete case data to results from 5 imputed datasets with imputed variables from multiple imputations.
- **Supplementary Table 2.** Demographic and clinical characteristics of the study population after propensity score matching.
- **Supplementary Table 3.** Sensitive analysis of the association between living arrangements and hypertension.
- **Supplementary Table 4.** Association between living arrangement transitions and hypertension
- **Supplementary Figure 1.** Flowchart of the included study population.
- **Supplementary Figure 2.** The standardized mean differences (SMD) of the variables.

**Supplementary Table 1.** Distributions of Variables with Missing Data Comparing Observed Complete Case Data to Results from 5 Imputed Datasets with Imputed Variables from Multiple Imputations

| Characteristic | Missing Data, no. (%) | Complete Case | Multiple Imputation1 | Multiple Imputation2 | Multiple Imputation3 | Multiple Imputation4 | Multiple Imputation5 |
| --- | --- | --- | --- | --- | --- | --- | --- |
| Married, no. (%) | 6(0.07) | 2837(33.9) | 2952(33.6) | 2952(33.6) | 2954(33.6) | 2953(33.6) | 2951(33.6) |
| Education (year), no. (%) | 25(0.28) |  |  |  |  |  |  |
| 0 |  | 5236(62.6) | 5532(63.0) | 5531(63.0) | 5537(63.0) | 5535(63.0) | 5534(63.0) |
| ≥1 |  | 3124(37.4) | 3250(37.0) | 3251(37.0) | 3245(37.0) | 3247(37.0) | 3248(37.0) |
| Smoker, no. (%) | 9(0.10) |  |  |  |  |  |  |
| Never |  | 5522(66.1) | 5813(66.2) | 5810(66.2) | 5811(66.2) | 5811(66.2) | 5811(66.2) |
| Current |  | 1564(18.7) | 1625(18.5) | 1626(18.5) | 1627(18.5) | 1626(18.5) | 1626(18.5) |
| Former |  | 1274(15.2) | 1344(15.3) | 1346(15.3) | 1344(15.3) | 1345(15.3) | 1345(15.3) |
| Drinker, no. (%) | 20(0.23) |  |  |  |  |  |  |
| Never |  | 5644(67.5) | 5950(67.8) | 5949(67.7) | 5957(67.8) | 5952(67.8) | 5951(67.8) |
| Current |  | 1587(19.0) | 1638(18.7) | 1639(18.7) | 1634(18.6) | 1637(18.6) | 1638(18.7) |
| Former |  | 1129(13.5) | 1194(13.6) | 1194(13.6) | 1191(13.6) | 1193(13.6) | 1193(13.6) |
| Regular exercise, no. (%) | 21(0.24) |  |  |  |  |  |  |
| Never |  | 5112(61.1) | 5389(61.4) | 5386(61.3) | 5386(61.3) | 5389(61.4) | 5388(61.4) |
| Current |  | 2258(27.0) | 2340(26.6) | 2342(26.7) | 2345(26.7) | 2342(26.7) | 2343(26.7) |
| Former |  | 990(11.8) | 1053(12.0) | 1054(12.0) | 1051(12.0) | 1051(12.0) | 1051(12.0) |
| Total sleep time (h), no. (%) | 30(0.34) |  |  |  |  |  |  |
| <6 |  | 982(11.7) | 1041(11.9) | 1040(11.8) | 1041(11.9) | 1041(11.9) | 1039(11.8) |
| 6-9 |  | 4297(51.4) | 4491(51.1) | 4494(51.2) | 4495(51.2) | 4493(51.2) | 4497(51.2) |
| >9 |  | 3081(36.9) | 3250(37.0) | 3248(37.0) | 3246(37.0) | 3248(37.0) | 3246(37.0) |
| Annual household income (yuan), no. (%) | 104(1.18) |  |  |  |  |  |  |
| <10000 |  | 3422(40.9) | 3604(41.0) | 3603(41.0) | 3597(41.0) | 3599(41.0) | 3601(41.0) |
| 10000-30000 |  | 2837(33.9) | 2954(33.6) | 2947(33.6) | 2948(33.6) | 2946(33.5) | 2956(33.7) |
| >30000 |  | 2101(25.1) | 2224(25.3) | 2232(25.4) | 2237(25.5) | 2237(25.5) | 2225(25.3) |
| BMI (kg/m^2^), median (IQR) | 158(1.80) | 19.56(17.71,21.93) | 19.59(17.71,21.95) | 19.59(17.72,21.95) | 19.57(17.71,21.94) | 19.57(17.71,21.95) | 19.59(17.71,21.97) |
| Diabetes, no. (%) | 19(0.22) | 141(1.7) | 144(1.6) | 145(1.7) | 144(1.6) | 144(1.6) | 145(1.7) |
| Heart disease, no. (%) | 17(0.19) | 520(6.2) | 555(6.3) | 555(6.3) | 555(6.3) | 554(6.3) | 553(6.3) |
| Stroke or CVD, no. (%) | 21(0.24) | 364(4.4) | 411(4.7) | 413(4.7) | 415(4.7) | 413(4.7) | 413(4.7) |
| Respiratory disease, no. (%) | 18(0.21) | 917(11.0) | 964(11.0) | 963(11.0) | 962(11.0) | 963(11.0) | 963(11.0) |
| Cancer, no. (%) | 30(0.34) | 36(0.4) | 40(0.5) | 40(0.5) | 40(0.5) | 40(0.5) | 41(0.5) |

*Notes*: IQR = interquartile range; CVD = [cardiovascular diseases](https://www.who.int/news-room/fact-sheets/detail/cardiovascular-diseases-(cvds)).

**Supplementary Table 2.** Demographic and Clinical Characteristics of the Study Population after Propensity Score Matching

| Characteristics | Overall (n =2360) | Living With Family (n=1180) | Living Alone (n=1180) | P value |
| --- | --- | --- | --- | --- |
| Age (years), median (IQR) | 89.00 (81.00,95.00) | 89.00 (80.75,95.00) | 89.00 (81.00,94.00) | 0.324 |
| Female, no. (%) | 1437(60.9) | 727(61.6) | 710(60.2) | 0.500 |
| Married, no. (%) | 147(6.2) | 73(6.2) | 74(6.3) | 1.000 |
| Urban area, no. (%) | 775(32.8) | 390(33.1) | 385(32.6) | 0.861 |
| Education (year), no. (%) | | | | 0.444 |
| 0 | 1660(70.3) | 839(71.1) | 821(69.6) |  |
| ≥1 | 700(29.7) | 341(28.9) | 359(30.4) |  |
| Smoking status, no. (%) | | | | 0.773 |
| Never | 1648(69.8) | 832(70.5) | 816(69.2) |  |
| Current | 409(17.3) | 200(16.9) | 209(17.7) |  |
| Former | 303(12.8) | 148(12.5) | 155(13.1) |  |
| Drinking status, no. (%) | | | | 0.577 |
| Never | 1652(70.0) | 833(70.6) | 819(69.4) |  |
| Current | 407(17.2) | 205(17.4) | 202(17.1) |  |
| Former | 301(12.8) | 142(12.0) | 159(13.5) |  |
| Regular exercise, no. (%) | | | | 0.124 |
| Never | 1554(65.8) | 800(67.8) | 754(63.9) |  |
| Current | 601(25.5) | 286(24.2) | 315(26.7) |  |
| Former | 205(8.7) | 94(8.0) | 111(9.4) |  |
| ADL limitation | 261(11.1) | 134(11.4) | 127(10.8) | 0.694 |
| Annual household income (yuan), no. (%) | | | | 0.478 |
| <10000 | 1559(66.1) | 785(66.5) | 774(65.6) |  |
| 10001-30000 | 466(19.7) | 222(18.8) | 244(20.7) |  |
| >30000 | 335(14.2) | 173(14.7) | 162(13.7) |  |
| Sleep time (h), no. (%) |  |  |  | 0.582 |
| <6 | 349(14.8) | 182(15.4) | 167(14.2) |  |
| 6–9 | 1162(49.2) | 583(49.4) | 579(49.1) |  |
| ≥9 | 849(36.0) | 415(35.2) | 434(36.8) |  |
| BMI (kg/m^2^), no. (%) | | | | 0.824 |
| Underweight (<18.5) | 894(37.9) | 458(38.8) | 436(36.9) |  |
| Normal (18.5–23.9) | 1223(51.8) | 601(50.9) | 622(52.7) |  |
| Overweight (24–27.9) | 189(8.0) | 94(8.0) | 95(8.1) |  |
| Obese (≥28) | 54(2.3) | 27(2.3) | 27(2.3) |  |
| Diabetes, no. (%) | 35(1.5) | 18(1.5) | 17(1.4) | 1.000 |
| Heart disease, no. (%) | 135(5.7) | 63(5.3) | 72(6.1) | 0.478 |
| Stroke or CVD, no. (%) | 85(3.6) | 45(3.8) | 40(3.4) | 0.659 |
| Respiratory disease, no. (%) | 243(10.3) | 123(10.4) | 120(10.2) | 0.892 |
| Cancer, no. (%) | 7(0.3) | 5(0.4) | 2(0.2) | 0.449 |

*Notes*: IQR = interquartile range; ADL = activities of daily living; CVD = [cardiovascular diseases](https://www.who.int/news-room/fact-sheets/detail/cardiovascular-diseases-(cvds)).

**Supplementary Table 3.** Sensitive Analysis of the Association between Living Arrangements and Hypertension

| Variables | Unadjusted Model  HR (95% CI) | Model 1  HR (95% CI) | Model 2  HR (95% CI) |
| --- | --- | --- | --- |
| Competing risk model (n = 8782) | | | |
| Living with family | Reference | Reference | Reference |
| Living alone | 1.25 (1.14-1.37) | 1.23 (1.11-1.37) | 1.23 (1.11-1.37) |
| After propensity score matching (n = 2360) | | | |
| Living with family | Reference | Reference | Reference |
| Living alone | 1.24 (1.07-1.42) | 1.26 (1.09-1.45 | 1.27 (1.10-1.47) |
| Excluded participants who were dead during follow-up (n = 5271) | | | |
| Living with family | Reference | Reference | Reference |
| Living alone | 1.11 (1.00-1.24) | 1.17 (1.03-1.32) | 1.17 (1.04-1.33) |
| Excluded participants who suffered from hypertension in the first 1 year of follow-up (n = 8621) | | | |
| Living with family | Reference | Reference | Reference |
| Living alone | 1.14 (1.03-1.26) | 1.20 (1.07-1.34) | 1.20 (1.07-1.35) |
| Excluded participants who had diabetes, heart disease, stroke, CVD, respiratory disease, or cancer (n = 6957) | | | |
| Living with family | Reference | Reference | Reference |
| Living alone | 1.12 (1.01-1.25) | 1.16 (1.02-1.31) | 1.16 (1.02-1.31) |

*Notes*: HR = hazard ratio; CI = confidence interval.

Model 1 was adjusted for age, gender, [educational](javascript:;) [level](javascript:;), residence, marital status, annual household income, drinking status, smoking status, regular exercise, and activities of daily living limitation.

Model 2 was further adjusted for sleep time, [body mass index](javascript:;), diabetes, heart disease, stroke, [cardiovascular diseases](https://www.who.int/news-room/fact-sheets/detail/cardiovascular-diseases-(cvds)), respiratory disease, and cancer.

**Supplementary Table 4.** Association between Living Arrangement Transitions and Hypertension

| Variables | Unadjusted Model | | Model 1 | | Model 2 | |
| --- | --- | --- | --- | --- | --- | --- |
|  | HR (95% CI) | P value | HR (95% CI) | P value | HR (95% CI) | P value |
| Not alone/Not alone (n=3869) | Reference |  | Reference |  | Reference |  |
| Not alone/Alone (n=406) | 1.09 (0.94-1.27) | 0.259 | 1.09 (0.94-1.27) | 0.263 | 1.07 (0.92-1.25) | 0.364 |
| Alone/Not alone (n=354) | 1.09 (0.93-1.28) | 0.300 | 1.11 (0.94-1.32) | 0.221 | 1.12 (0.94-1.32) | 0.197 |
| Alone/Alone (n=497) | 1.18 (1.03-1.36) | 0.019 | 1.25 (1.07-1.46) | 0.005 | 1.24 (1.06-1.45) | 0.006 |

*Notes*: HR = hazard ratio; CI = confidence interval.

Model 1 was adjusted for age, gender, [educational](javascript:;) [level](javascript:;), residence, marital status, annual household income, drinking status, smoking status, regular exercise, and activities of daily living limitation.

Model 2 was further adjusted for sleep time, [body mass index](javascript:;), diabetes, heart disease, stroke, [cardiovascular diseases](https://www.who.int/news-room/fact-sheets/detail/cardiovascular-diseases-(cvds)), respiratory disease, and cancer.


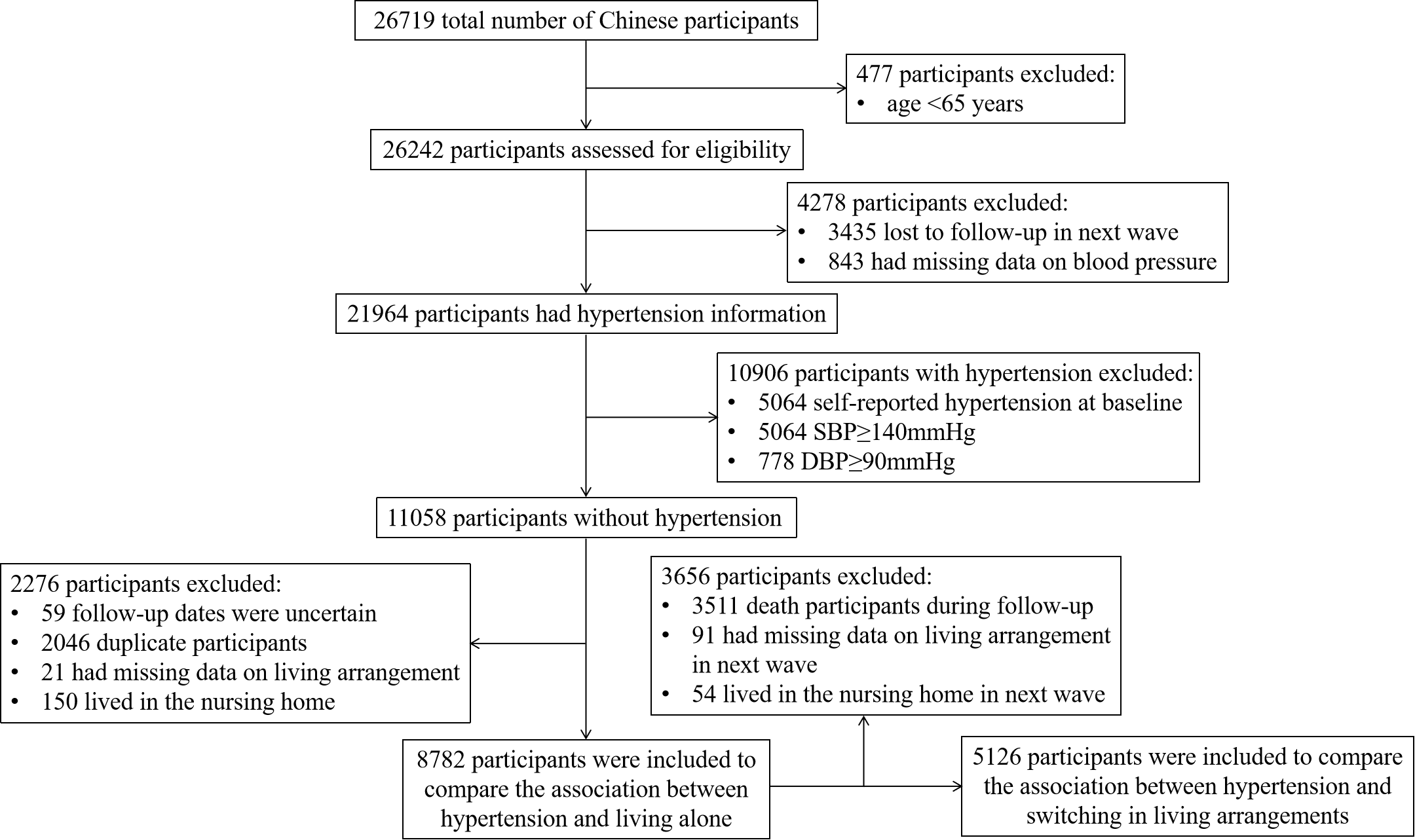


**Supplementary Figure 1.** Flowchart of the Included Study Population


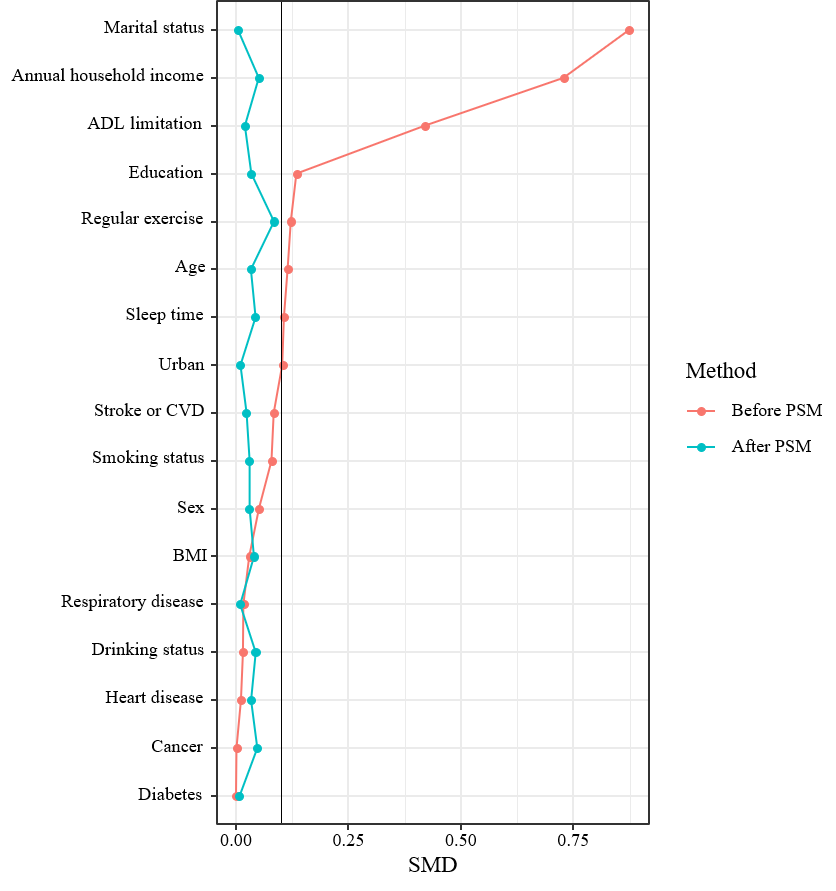


**Supplementary Figure 2.** The Standardized Mean Differences (SMD) of the Variables.

*Notes*: ADL = activities of daily living; CVD = [cardiovascular diseases](https://www.who.int/news-room/fact-sheets/detail/cardiovascular-diseases-(cvds)); PSM = propensity score matching.
